# Gp91^phox ^(NOX2) in classically activated microglia exacerbates traumatic brain injury

**DOI:** 10.1186/1742-2094-7-41

**Published:** 2010-07-26

**Authors:** Kenji Dohi, Hirokazu Ohtaki, Tomoya Nakamachi, Sachiko Yofu, Kazue Satoh, Kazuyuki Miyamoto, Dandan Song, Shohko Tsunawaki, Seiji Shioda, Tohru Aruga

**Affiliations:** 1 Department of Emergency and Critical Care Medicine, Showa University School of Medicine, Shinagawa-Ku, Tokyo 142-8555, Japan; 2 Department of Anatomy, Showa University School of Medicine, Shinagawa-Ku, Tokyo 142-8555, Japan; 3 Department of Infectious Diseases, National Research Institute for Child Health and Development, Setagaya-ku, Tokyo, 157-8535, Japan

## Abstract

**Background:**

We hypothesized that gp91^phox ^(NOX2), a subunit of NADPH oxidase, generates superoxide anion (O_2_^-^) and has a major causative role in traumatic brain injury (TBI). To evaluate the functional role of gp91^phox ^and reactive oxygen species (ROS) on TBI, we carried out controlled cortical impact in gp91^phox ^knockout mice (gp91^phox-/-^). We also used a microglial cell line to determine the activated cell phenotype that contributes to gp91^phox ^generation.

**Methods:**

Unilateral TBI was induced in gp91^phox-/- ^and wild-type (Wt) mice (C57/B6J) (25-30 g). The expression and roles of gp91^phox ^after TBI were investigated using immunoblotting and staining techniques. Levels of O_2_^- ^and peroxynitrite were determined *in situ *in the mouse brain. The activated phenotype in microglia that expressed gp91^phox ^was determined in a microglial cell line, BV-2, in the presence of IFNγ or IL-4.

**Results:**

Gp91^phox ^expression increased mainly in amoeboid-shaped microglial cells of the ipsilateral hemisphere of Wt mice after TBI. The contusion area, number of TUNEL-positive cells, and amount of O_2_^- ^and peroxynitrite metabolites produced were less in gp91^phox-/- ^mice than in Wt. In the presence of IFNγ, BV-2 cells had increased inducible nitric oxide synthase and nitric oxide levels, consistent with a classical activated phenotype, and drastically increased expression of gp91^phox^.

**Conclusions:**

Classical activated microglia promote ROS formation through gp91^phox ^and have an important role in brain damage following TBI. Modulating gp91^phox ^and gp91^phox ^-derived ROS may provide a new therapeutic strategy in combating post-traumatic brain injury.

## Background

Traumatic brain injury (TBI) is a serious condition in emergency medicine, and its pathophysiological profile is varied and complicated. One of the neurotoxic factors thought to be involved is oxidative stress [1,2]. A large number of studies have reported that oxidative stress, which generates reactive oxygen species (ROS), plays a key role in the development of TBI [1,3,4]. Consequently, one of the most obvious ways to manage TBI may be to control ROS generation [1] given that animal experiments have supported the notion that free radical scavengers and antioxidants dramatically reduce cerebral damage [1,5,6]. The superoxide anion (O_2_^-^) is an important free radical, and is the source of other ROS that lead to lipid peroxidation [7]. Cyclooxygenase, xanthine oxidase, and NADPH oxidases of the NOX family are well known generators of O_2_^- ^in the brain. However, the main cellular mediator of O_2_^- ^generation after TBI has not yet been determined. NADPH oxidase, a multiunit enzyme initially discovered in neutrophils, has recently emerged as a major generator of ROS in neurons, glial cells and cerebral blood vessels [8-10]. NADPH oxidase is composed of membrane-bound (p22^phox ^and gp91^phox^) and cytoplasmic subunits (p40^phox^, p47^phox^, and p67^phox^). Several homologs of the catalytic subunit of the enzyme, gp91^phox^, also termed NOX2, exist (NOX1 through NOX5) [11,12]. It has been reported that gp91^phox^-containing NADPH oxidase produces a large amount of O_2_^- ^in leukocytes, while numerous papers have reported on the role for gp91^phox ^in various neurodegenerative conditions [13,14]. However, the source and the roles of gp91^phox ^after TBI have not been established. In this study, we used the gp91^phox-/- ^mouse to investigate the kinetics and the roles of gp91^phox ^following TBI.

## Methods

### Animals

All experimental procedures involving animals were approved by the Institutional Animal Care and Use Committee of Showa University. The gp91^phox-/- ^(C57/B6J) mice are described by Dinauer et al. [15], Wild mice (Wt) were generated from the same chimeric founder, and experiments were performed in age- and weight-matched animals.

### Controlled cortical impact model

Mice were anesthetized with 2% sevoflurane in 70% N_2_O and 30% O_2_. A controlled cortical impact was made using a pneumatically controlled impactor device as described previously [16].

### Cell culture

We used the BV-2 microglial cell line to investigate which microglia cells express gp91^phox ^[17]. This mouse BV-2 cell line was obtained from Interlab Cell Line Collection (Genova, Italy) and cultured in 10% RPMI1640 (RPMI1640 with 10% fetal calf serum [FCS], 100 U/ml penicillin, 100 μg/ml streptomycin, and 2 mM L-glutamine [all from GIBCO/BRL, Grand Island, NY]). The cells were grown at 37°C in a humidified 5% CO_2 _incubator. After harvesting, the cells were washed with PBS twice and resuspended with experimental medium (Dulbecco's modified Eagle's medium [GIBCO/BRL] with 1% FCS, 100 U/ml penicillin, 100 μg/ml streptomycin, and 2 mM L-glutamine). The cells were seeded into six-well plates at 1 × 10^6 ^cells/well/ml and then exposed to IFNγ (10 ng/ml), IL-4 (20 ng/ml), IL-10 (10 ng/ml, all from Peprotech, Rocky Hill, NJ), or vehicle (n = 3/group). Twenty-four hours later, the cells were collected by centrifugation. The samples were kept at -30°C until analysis.

### Western blot analysis

The cerebrum was removed from decapitated animals at 0 (sham-operated), 24, and 48 hours after TBI and divided into the ipsilateral and contralateral hemispheres. These samples were then homogenized in lysis buffer (10 mM Tris-HCl [pH 7.4], 0.15 M NaCl and 1% Triton X-100, 1 mM EGTA, 50 mM NaF, 2 mM sodium orthovanadate, 10 mM sodium pyrvate, and protease inhibitor cocktail [Sigma, St. Louis, MO]), and centrifuged at 12,000 × *g *for 10 minutes on ice. BV-2 samples were sonicated for 10 seconds with lysis buffer to prepare cell suspensions.

After determination of the protein concentration (BCA protein assay, Thermo Fisher Scientific, Waltham, MA), appropriate amounts of samples were electrophoresed. The separated proteins were then transferred to polyvinylidinene fluoride membranes (Bio-Rad, Hercules, CA). After blocking with 2% Blockace (DS Pharma, Osaka, Japan), the membranes were probed with primary antibodies. After washing, the membrane was probed with horseradish peroxidase (HRP)-conjugated secondary antibodies. The protein bands were detected by chemiluminescence (SuperSignal West Dura Extended Duration Substrate; Pierce, Rockford, IL) and exposed onto X-ray film. The films were scanned, and the signal densities were quantified using the UN-SCAN-IT gel analysis program (Silk Scientific, Orem, UT).

### Immunohistochemistry

Mice subjected to TBI were placed under pentobarbital (50 mg/kg, i.p.) anesthesia and perfused with 0.9% NaCl followed by 2% paraformaldehyde (PFA). Brains were removed and processed to frozen blocks that were then cut into 8-μm sections (*n *= 4-5/group).

The sections were incubated with 0.3% H_2_O_2 _and then incubated with PBS containing 5% normal horse serum to mask nonspecific reactions. Next, the sections were incubated with antibody raised against gp91^phox ^[18]. One day later, the sections were rinsed and incubated with biotinylated goat anti-rabbit IgG (Santa Cruz Biotechnology, Santa Cruz, CA), and then with an avidin-biotin complex solution (Vector Laboratories, Burlingame, CA) followed by diaminobenzidine (DAB; Vector) as a chromogen.

A similar procedure was used for multiple immunostaining, except that the sections were not incubated with 0.3% H_2_O_2_, and were incubated with Alexa-labeled fluorescence secondary antibodies. Primary and secondary antibodies for multiple-staining are listed in Table 1. 4,6-Diamidine-2-phenylindole dihydrochloride (DAPI, 1:10,000; Roche, Mannheim, Germany) was used for nuclear staining. The fluorescence and immunolabeling were detected using a confocal laser microscope (AX-10, Zeiss; Oberkochen, Germany).

**Table 1:** Antibodies used for immunoblotting (IB) and immunohistochemistry (IHC)

Antibody	Antigen	Host	Company	Catalog #	Dilution
***Primary antibody (clone #)***					
gp91^phox^	Human gp91	Rabbit	See ref 18		4,000 (IB) 200 (IHC)
p22^phox^	Human p22	Rabbit	See ref 18		3,000
iNOS	Mouse iNOS	Rabbit	Transduction Laboratories (Lexington, KY)	N32030	10,000
Ym1	Mouse Ym1	Rabbit	StemCell Tech (Vancouver, BC, Canada)	01404	1,000
GAPDH (6C5)	Rabbit GAPDH	Mouse	Chemicon International (Temecula, CA)	MAB374	3,000
β-Actin (AC-74)	Mouse β-Actin	Mouse	Sigma (St Louise, MO)	A5316	4,000
CD11b (5C6)	Mouse CD11b	Rat	Serotec (Oxford, UK)	MCA711	500
GFAP (G-A-5)	Mouse GFAP	Mouse	Sigma (St Louise, MO)	G3893	1000
NeuN	Mouse NeuN	Mouse	Chemicon International (Temecula, CA)	MAB377	1000
3-NT	Nitrated KLH	Rabbit	Upstate Biotechnology (Lake Placid, NY)	06-284	100
***Secondary antibody (conjugation)***					
Mouse IgG (HRP)	Mouse IgG	Sheep	GE Healthcare Bioscience (Little Chalfont, UK)	NA931	2,000
Rabbit IgG (HRP)	Rabbit IgG	Donkey	GE Healthcare Bioscience (Little Chalfont, UK)	NA934	3,000
Rabbit IgG (biotinylated)	Rabbit IgG	Goat	Santa Cruz Biotechnology (Santa Cruz, CA)	SC-2040	200
Mouse IgG (Alexa 546)	Mouse IgG	Goat	Molecular Probes (Eugene, OR)	A11030	400
Rabbit IgG (Alexa 488 or 546)	Rabbit IgG	Goat	Molecular Probes (Eugene, OR)	A11034 or 11035	400
Rat IgG (Alexa 546)	Rat IgG	Goat	Molecular Probes (Eugene, OR)	A11081	400

### Evaluation of the injured brain area

The areas of injured brain were determined using 2,3,5-triphenyltetrazolium chloride (TTC) staining of tissues 48 hours after TBI. The animals were decapitated, and the brain was sectioned into four 2-mm coronal sections by using a mouse brain matrix. The brain slices were then stained with 2% TTC at 37°C for 30 min and photographed on the anterior surface of each section with a scale bar. The areas of injured brain were delineated by examining differences between the ipsilateral and contralateral regions in the center slice of injured brain and measured by using NIH Image software. http://rsb.info.nih.gov/nih-image/about.html.

### Evaluation of apoptosis-like cell death

To determine neural apoptosis-like cell death, terminal deoxynucleotidyl transferase-mediated dUTP end-labeling (TUNEL) staining (In Situ Cell Death Detection Kit, POD; Roche) was performed 48 hours after TBI (*n *= 5) and the number of TUNEL-positive cells in the ipsilateral hemisphere was then counted and compared gp91^phox-/- ^with Wt mice in a similar cortical region (40 × magnification).

### In situ detection of O_2_^-^

Production of O_2_^- ^was determined by *in situ *detection of oxidized hydroethidium (HEt) [19]. With the animal placed under anesthesia, the HEt solution was administered (1 mg/mL 0.9% NaCl with 1% DMSO) into the jugular vein (*n *= 3 per group) 48 hours after TBI. Fifteen minutes later, the brain was removed and frozen in blocks and cryosectioned (8 μm) in the coronal plane. To demonstrate the cellular distribution of Et, the sections were co-stained with antibodies raised against gp91^phox^, CD11b, GFAP, or NeuN. Fluorescence was detected using confocal laser microscopy (AX-10, Zeiss, Germany)

### NO and TNFα measurement in media

Levels of NO and TNFα production are markers of classically activated microglia [20]. NO production was measured using the Griess method (Dojindo, Kumamoto, Japan) as total NO (NO_2_^- ^and NO_3_^-^). TNFα production was measured by enzyme-linked immunosorbent assay using the Duoset ELISA Development System (R&D Systems, Minneapolis, MN).

### Assay for arginase activity

Arginase is a marker for alternatively activated microglia [20]. Arginase activity was measured according to a previous paper [21] with minor modification. In brief, the cell homogenate was mixed with equal volumes of prewarmed 50 mM Tris-HCl, pH 7.5 containing 10 mM MnCl_2 _and incubated for 15 minutes at 55°C. The mixture was incubated in 0.25 M L-arginine for 60 minutes at 37°C to produce urea from arginine and the reactions were stopped by adding Stop solution (H_2_SO_4_/H_3_PO_4_/H_2_O, 1:3:7). Then, 1% (final concentration) 1-phenyl-1, 2-propanedione-2-oxime (ISPF) in ethanol was added to the solution, which was heated at 100°C for 45 min. The reaction between urea and ISPF produced a pink color, and absorption was measured at 540 nm.

### Statistical analysis

Data are expressed as mean ± SE for *in vivo *experiments. Data are expressed as mean ± SD for *in vitro *experiments Statistical comparisons were performed using the Student's *t *tests and two-way analysis of variance (ANOVA) as appropriate. *P *values less than 0.05 were considered statistically significant.

## Results

### Gp91^phox ^is upregulated in the peri-contusional region after traumatic brain injury

**Figure 1:**
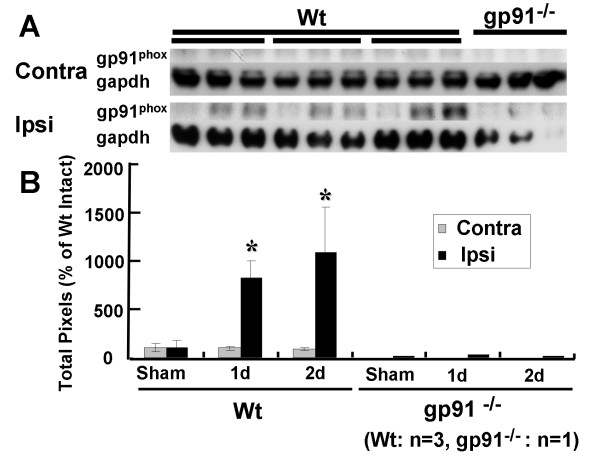
**Characterization of gp91^phox ^after traumatic brain injury (TBI) using western immunoblots**. (A) Immunoblotting signals before and after TBI in wild-type (Wt) mice (left) and gp91^phox-/- ^mice (right). (B) In Wt mice, gp91^phox ^levels were significantly increased on day 1 and day 2 after TBI (*p < 0.05 relative to sham). Gp91^phox ^levels on the ipsilateral side were greater than on the contralateral side on day 1 and day 2 after TBI (*p < 0.05). Each value is the mean ± SE (n = 3). Note that the intensity of each sample was quantified and corrected relative to the labeling control, actin. Increasing levels of gp91^phox ^were not seen in gp91^phox-/- ^mice.

Fig 1 shows the results of immunoblotting experiments to describe the expression of gp91^phox ^after TBI. Protein levels of gp91^phox ^were not increased after TBI in the contralateral hemisphere of Wt mice. In the ipsilateral hemisphere of Wt mice, protein levels of gp91^phox ^were greater at 1 and 2 days after TBI relative to the contralateral hemisphere (Fig 1A, B). The data indicate that gp91^phox ^increased in the ipsilateral hemisphere following TBI stress.

**Figure 2:**
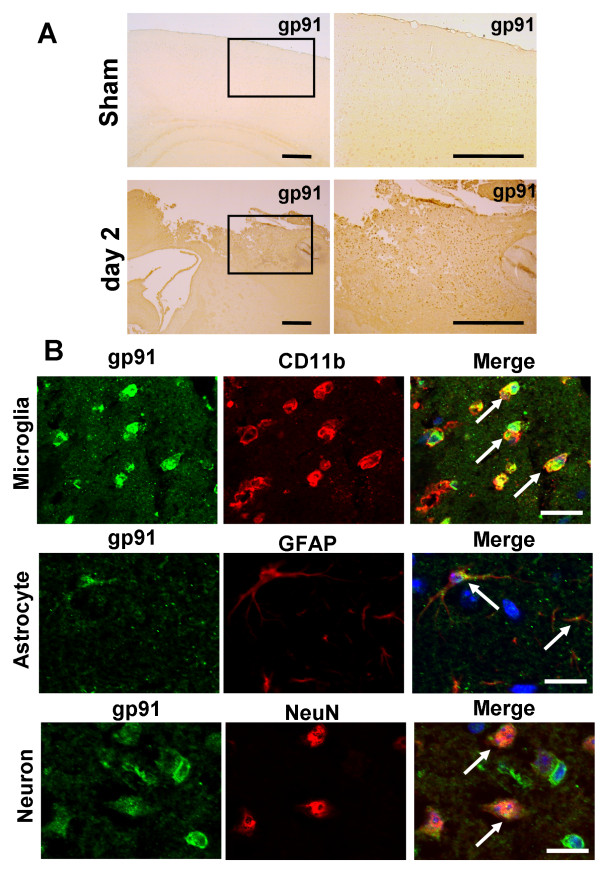
**Expressions and cell identification of gp91^phox ^in peri-contusional area after traumatic brain injury (TBI)**. (A) In sham-operated animals, weak immunoreactivity for gp91^phox ^was observed in the cortex (upper panel). Two days after TBI, gp91^phox ^immunoreactivity was dramatically increased in the peri-contusional area (lower panel). Scale bars = 400 μm. (B) Co-immunostaining of gp91^phox ^with cell markers in the peri-contusional region. Immunostaining was carried out using antibodies for gp91^phox ^(shown in green) together with Integrin alpha M (CD11b) (upper), glial fibrillary acidic protein (GFAP) (middle), and neuronal nuclear antigen (NeuN) (lower). Microglia, astrocyte, and neuron markers are shown in red. Gp91^phox ^immunoreactive cells were co-labeled with all cell markers. Particularly strong expression of gp91^phox ^was detected in microglial-like cells (CD11b-positive cells). Cells were counter-stained with DAPI to show nuclei (blue). Scale bars = 20 μm.

The results of single immunohistochemical detection of gp91^phox ^after TBI are presented Fig 2A. Two days after TBI, gp91^phox ^immunoreactivity was dramatically increased in the peri-contusion region.

### Gp91^phox ^is mainly expressed by cytotoxic-type classically activated microglia in the peri-contusional regions after TBI

To identify the cell types expressing gp91^phox ^in the peri-contusional area, cells were co-labelled with antibodies raised against gp91^phox ^and markers of microglial (CD11b), astroglial (GFAP), and neuronal (NeuN) cells (Fig 2B). The immunoreactivity for gp91^phox ^was mainly co-localized to microglia that had amoeboid-like morphological features with round cell body and short processes, suggesting cytotoxic-type classical activation. Immunoreactive staining for gp91^phox ^was also co-localized to a few astrocytes and neurons.

### Gp91^phox ^inhibition reduces the severity of TBI *in vivo*

**Figure 3:**
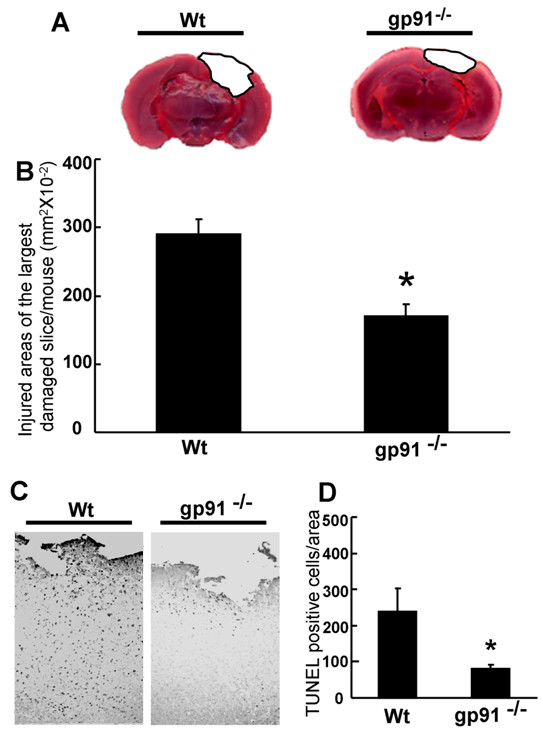
**Brain damage and cell death after traumatic brain injury in wild type (Wt) mice and gp91^phox-/- ^mice**. (A) 2,3,5-triphenyltetrazolium chloride (TTC)-stained coronal brain sections from Wt mice (left) and gp91^phox-/- ^mice (right) 2 days after TBI. (B) The contusion area in gp91^phox-/- ^mice (170.5 ± 71.5 mm^2 ^× 10^-2^, n = 20) was significantly smaller than that in the Wt group (290.7 ± 94.0 mm^2 ^× 10^-2^, n = 10, *p < 0.01, t test). (C) Terminal deoxynucleotidyl transferase dUTP nick end labeling (TUNEL)-staining in peri-contusional area of wild type (left) and gp91^phox-/- ^(right) mice 48 hours after TBI. TUNEL-positive cells numbers in the gp91^phox-/- ^mice (117 ± 69.3 cells/area, n = 6) were significantly smaller than those in the Wt group (252 ± 128.9 cells/area, n = 6, *p < 0.05, t test) (D). All values represent the mean ± SE.

TTC staining was used to evaluate the role of gp91^phox ^on the severity of TBI in gp91^phox-/- ^and Wt mice (Fig 3A, B). Images of the TTC-stained anterior surface of coronal sections demonstrated that the injured brain area in gp91^phox-/- ^mice was significantly smaller (*P *< 0.01) than that of the Wt mice (Fig. 3B). We verified the prevention of the cortical injury with TUNEL staining, which was used to identify apoptotic-like cell death at 48 hours after TBI (Fig 3C, D). The number of TUNEL-positive cells of gp91^phox-/- ^mice was significantly less (*p *< 0.05) compared with Wt mice (Fig 3D).

### Gp91^phox ^gene deletion reduces superoxide radical (O_2_^-^) production after TBI

**Figure 4:**
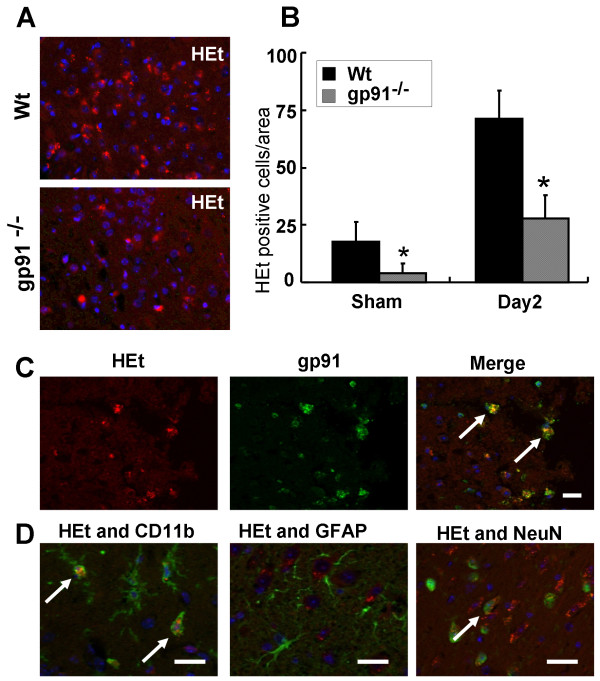
**The roles of gp91^phox ^in superoxide radical production after traumatic brain injury)**. (A) Superoxide radical production (hydroethidium [HEt]-positive cells: shown in red) in the peri-contusional area of wild type (upper) and gp91^phox-/- ^(lower) mice 2 days after TBI. (B) TBI-induced superoxide radical production (HEt-positive cells) in the peri-contusional area was significantly attenuated by gp91^phox ^deficiency in sham-operated mice, and 2 days after TBI (*p < 0.05, t test). All values represent mean ± SE. (C, D) Co-localization of HEt-positivity with cell markers in the peri-contusional region. Cell identification was carried out using antibodies for gp91^phox ^(C), CD11b (D, left), GFAP (D, middle), and Neu N (D, right) (shown in green). HEt-positive cells were co-localized with gp91^phox^-positive cells (C, right, arrows) and microglia-like cells (D, left, arrows). Although HEt-positive cells were also slightly co-localized with neurons (D, right, arrows), immunoreactivity was lower than for gp91^phox^-positive cells and microglia-like cells. Cells were counter-stained with DAPI to show nuclei (blue). Scale bars = 20 μm (C, D).

High intensity fluorescence staining by the oxidation product Et was observed, indicating high levels of O_2_^- ^production. The peri-contusional area of Wt mice accounted for much of the Et signal after TBI (Fig 4A). Et fluorescence was lower in gp91^phox-/- ^mice (Fig 4A) compared to Wt mice (*p *< 0.05) (Fig 4B). The Et fluorescence was associated with p91^phox^-positive cells (Fig. 4C), and CD11b-positive microglial cells (Fig. 4D). Some Et fluorescence was slightly detected in a few neurons (Fig. 4D). These results suggest that O_2_^- ^is mainly produced by microglia expressing gp91^phox^.

### Gp91^phox ^gene deletion reduces 3-NT generation after TBI *in vivo*

**Figure 5:**
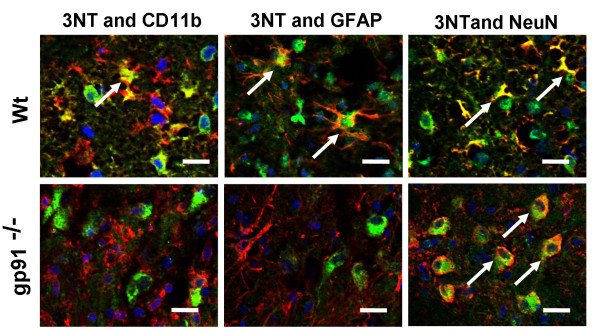
**Production and cell identification of peroxynitrite (ONOO^-^) in wild type (Wt) (upper) and gp91^phox-/- ^(lower) mice 48 hours after TBI**. ONOO^- ^in Wt mice was produced in microglia-like cells (left, arrows), astrocytes (middle, arrows), and degenerated neurons (right, arrows). In gp91^phox-/- ^mice, ONOO^- ^production was strongly suppressed. In neurons, only weak production of ONOO^- ^was observed, but degeneration of neurons was not seen (right, arrows). Scale bars = 20 μm.

Peroxynitrite (ONOO^-^) is an oxidant and nitrating agent and is synthesized by the reaction between O_2_^- ^and nitric oxide (NO). ONOO^- ^can damage a wide array of cellular molecules, including DNA and proteins. 3-NT is an oxidized metabolite of ONOO^- ^*in vivo*. We performed multiple-immunostaining for 3-NT and cell marker antibodies in the peri-contusional area at 48 hours. Immunoreactivity for 3-NT in Wt mice clearly co-localized with microglia, astrocytes, and degenerated neurons (Fig 5; upper panel). In gp91^phox-/- ^mice, however, 3-NT immunoreactions were dramatically suppressed in the microglial and astroglial cells (Fig 5). While 3-NT immunoreactivity was observed in gp91^phox-/- ^mouse neurons, the integrity of these cells was preserved (Fig 5). Taken together these findings suggest that production of O_2_^- ^and ONOO^- ^by gp91^phox ^may influence the severity of TBI.

### Classically activated BV-2 increased gp91^phox ^and p22^phox^

**Figure 6:**
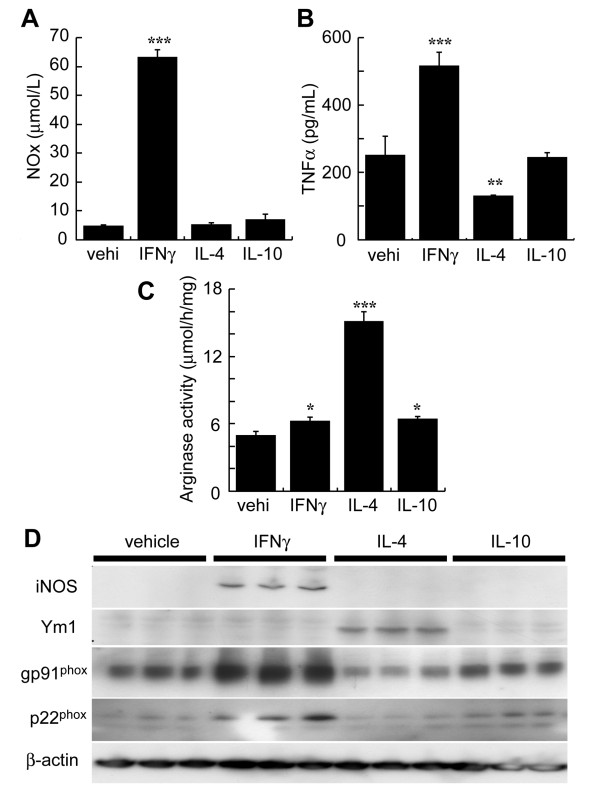
**Expression of gp91^phox ^and p22^phox ^in IFNγ-exposed classically activated mouse microglial BV-2 cells**. BV-2 cells were exposed to vehicle (vehi), IFNγ, IL-4, or IL-10. The levels of microglial phenotype markers (NOx, TNFα, arginase activity, iNOS, and Ym1), gp91^phox^, and p22^phox ^were evaluated in medium or cell homogenates 24 hours later. Production of (A) NOx and (B) TNFα were determined in the media. (C) Expression of iNOS, Ym1, gp91^phox^, and p22^phox ^was determined by immunoblotting with reduced samples. ß-Actin levels were used as an internal control. IFNγ-exposed BV-2 cells had increased NOx, TNFα, and iNOS levels, all of which are classical activating markers of the microglial phenotype. In contrast, IL-4-exposed BV-2 cells had increased arginase activity and Ym1 levels, which are alternative markers of activation. Gp91^phox ^and p22^phox ^levels were increased only in IFNγ-exposed BV-2 cells, suggesting induction by classically activated microglial cells.

From the results presented in Fig. 2 we suggested that gp91^phox ^co-localized with classical activated microglia. We then tried to identify the microglia that expressed gp91^phox ^by using the mouse microglial cell line BV-2 and by activating these cells with IFNγ, IL-4, or IL-10. As shown in Fig 6, the media from the BV-2 cells showed significantly increased total NO after exposure to IFNγ, but not to IL-4 or IL-10 at 24 hours. The IFNγ-exposed BV-2 cells had significantly increased TNFα in their media and expressed iNOS as well. All of these factors indicate that the BV-2 cells were activated according to the classically activated phenotype [20]. On the other hand, the IL-4-exposed BV-2 cells showed increased arginase activity and levels of Ym-1, indicating an alternatively activated phenotype [20]. IL-10-exposed BV-2 cells did not show any changes in levels of these factors. Immunoblotting experiments showed that gp91^phox ^levels increased in the IFNγ-exposed BV-2. Moreover, immunoblotting for a different NADPH oxidase subunit, p22^phox^, mirrored that of gp91^phox^, suggesting that not only gp91^phox ^but also NADPH oxidase expression was induced in the classically activated microglia.

## Discussions

We demonstrate here that gp91^phox ^is increased in the ipsilateral hemisphere after TBI and specifically in amoeboid-shaped microglial cells. Mice that are gene-deficient for gp91^phox ^exhibit reduced primary cortical damage, as evidenced by reduced areas of contusion, and reduced secondary damage as detected by TUNEL staining. Moreover, we have shown that the gene-deficient mice have lower levels of ROS at the injury site and widespread oxidative damage after TBI. Finally, we demonstrate in a BV-2 microglial cell line that gp91^phox ^and/or NADPH oxidase are increased in classically activated microglial cells which are activated by IFNγ.

Gp91^phox ^is expressed constitutively in neurons but not in glial cells, and O_2_^- ^production might play a role in neuronal homeostasis [22]. However, in neuropathological conditions including neurodegenerative diseases and stroke, the gp91^phox^-containing NADPH oxidase has been observed in glial cells, neurons, fibroblasts and vascular endothelial cells, and seems to be involved in ROS formation [22-29]. Microglial cell gp91^phox ^appears to be involved in the induction of neuronal damage in Parkinson's disease, Alzheimer's disease, and ischemic stroke [9,14,30,31]. In the present study, we demonstrated that gp91^phox ^is mainly expressed in microglia, and at lower levels in neurons and astrocytes. These microglia exhibit features of being classically activated. We then demonstrated that the microglial phenotypes expressed both gp91^phox ^and p22^phox^, which probably reflects the induction of NADPH oxidase in the BV-2 mouse microglial cell line that we used. Our results reveal that NADPH oxidase is induced in INFγ-stimulated, classically activated microglial cells characterized by increased NO, iNOS, and TNFα.

In gp91^phox-/- ^mice, *in situ *generation of O_2_^-^and an ONOO^- ^metabolite, 3-NT, were suppressed in microglia after TBI. ONOO^- ^produced by O_2_^- ^and NO after TBI [32] induces oxidative damage, secondary brain damage and neuroinflammation after TBI [5,33]. We have verified that TUNEL-positive apoptotic-like cells in the peri-contusional area and contusion area are suppressed in gp91^phox-/- ^mice. These results suggest that gp91^phox ^in classically activated microglia-like cells has a harmful role in primary and secondary brain damage after TBI.

The question of whether microglial cells play harmful or beneficial roles in CNS injures has been widely debated and reviewed over several decades [34-36]. The roles of activated microglia in neuroinflammation are thought to be complex. Classical activation is induced by IFNγ and is related to the production of proinflammatory mediators in the innate immune response. Another form of activation, called "alternative activation," is induced by IL-4 and IL-13 and, compared to classical activation, does not result in high levels of expression of proinflammatory mediators such as cytokines and NO. The roles of alternatively activated microglia during inflammatory process are thought to involve tissue repair, the production of anti-inflammatory cytokines, fibrosis, and extracellular matrix reconstruction. Recently, Ohtaki et al. reported that the injection of human mesenchymal stromal cells protects against ischemic brain injury by modulating inflammatory and immune responses through the alternative activation of microglia and/or macrophages [37]. These studies and our data suggest that controlling microglial activation and understanding its mechanism and functional significance following TBI may open exciting new therapeutic avenues.

The suppression of free radical generation and the scavenging of free radicals after brain damage are important therapies. Animal experiments have supported the notion that free radical scavengers and antioxidants dramatically reduce TBI [1,6,38]. Excessive O_2_^- ^may produce destructive hydroxyl radicals (OH^-^) and alkoxyl radicals (OR^-^) by the iron-catalyzed Haber-Weiss reaction. The brain is especially prone to radical damage because it is highly enriched in easily peroxidizable unsaturated fatty acid side chains and iron. Many studies investigating ischemic injury suggest that inhibition of NADPH oxidase or gp91 ^phox ^is an important therapeutic target for neuroprotection [39,40]. Some recent studies have demonstrated that expression of gp91^phox ^increases in brain after intracerebral hemorrhage, resulting in enhanced lipid peroxidation [24,41]. These studies also reported that hemorrhage volume, brain edema, and neurological function are reduced in gp91^phox-/- ^mice, while Lo et al. reported that neurological outcomes are improved in gp91^phox-/- ^mice [42]. On the other hand, Liu et al. reported that there are no significant differences in mortality rate, brain water content and intensity of oxidative stress between gp91^phox-/- ^and wild type mice in a mouse model of subarachnoid hemorrhage (SAH) [43]. In the present study, we have shown that, in gp91^phox-/- ^mice, gp91^phox ^expressed in classically-activated microglial-like cells plays a key role in O_2_^- ^production after TBI, and that gp91^phox^-derived O_2_^- ^is a key signal for contusion and cell death after TBI. Our findings indicate that gp91^phox ^inhibition and control of microglial classically-activation might provide a new therapeutic option by suppressing ROS generation after TBI. However, the indirect influence with TBI by which ROS production by other NOX families and immune cells such as endothelial cell or leukocytes is currently unclear and will be an important question for future TBI studies.

## Conclusions

In conclusion, we have shown that gp91^phox ^is expressed in classical activated microglial-like cells mainly in the peri-contusional area after TBI. An important generator of O_2_^- ^is gp91^phox ^during the acute phase of TBI. As ROS derived from gp91^phox ^play an important role in primary and secondary brain damage after TBI, modulation of gp91^phox ^in classically activated microglia and gp91^phox^-derived ROS may provide a new therapeutic strategy in combating post-traumatic brain injury.

## Competing interests

The authors declare that they have no competing interests.

## Authors' contributions

KD performed the majority of experiments and data analysis, and wrote the initial version of the manuscript. HO was involved in evaluation of microglia using BV-2 cell. TN, SY, DS and KM were substantial contributions to western blotting assay and immunohistochemistry. ST provided gp91 knockout mice. KS, SS and TA supervised all experimental procedures. All of the authors have read and approved the final version of the manuscripts.
